# Ketoacidosis associated with low-carbohydrate diet in a non-diabetic lactating woman: a case report

**DOI:** 10.1186/s13256-015-0709-2

**Published:** 2015-10-01

**Authors:** Louise von Geijer, Magnus Ekelund

**Affiliations:** 1 Department of Gynaecology & Obstetrics, Helsingborg Hospital, SE-251 87 Helsingborg, Sweden; 2 Department of Internal Medicine, Helsingborg Hospital, SE-251 87 Helsingborg, Sweden

**Keywords:** Diet, Ketoacidosis, Lactation, LCHF diet, Low-carbohydrate

## Abstract

**Introduction:**

Non-diabetic ketoacidosis is a rare condition which can be caused by starvation. Lack of glucose can force the body into ketogenesis causing a metabolic acidosis. As previously reported in the literature, ketoacidosis might, on rare occasions, be caused by a diet with low carbohydrate content. However, to the best of our knowledge this is the first reported case in the literature of ketoacidosis, in a non-diabetic patient, associated with a combination of low carbohydrate, high fat diet and lactation.

**Case presentation:**

A healthy non-diabetic, 32-year old white woman started a low carbohydrate, high fat diet when she was breastfeeding her son of 10 months of age. After 10 days she was admitted to our hospital with nausea and vomiting and a serum pH of 7.20 and base excess of −19. Clinical signs and blood samples were compatible with ketoacidosis. She was given fluids intravenously and insulin. No anamnestic or clinical signs of diabetes were found. She recovered quickly and was discharged 3 days later.

**Conclusions:**

Ketogenic diets like low carbohydrate, high fat may induce ketoacidosis. Lactation might further aggravate the condition and can perhaps even be the trigger into ketoacidosis. Health services should be aware of the risks associated with ketogenic diets, and be able to recognize this serious condition when it is presented.

## Introduction

Metabolic acidosis is a potentially life-threatening condition that can occur due to ketoacidosis caused by diabetes mellitus, starvation, alcohol ingestion or lactate acidosis, renal failure or ingestion of substances (that is, methanol, ethylene glycol or salicylate). Anamnesis, clinical signs and blood samples are used to determine the origin of the acidosis.

Ketosis during lactation is a well-known phenomenon in lactating cattle and is well described in veterinary literature [1]. During lactation the metabolic demand is higher in the lactating female due to milk production and secretion than in the non-lactating female [2]. The condition is rare since it is normally compensated for by increased dietary intake. Ketoacidosis not associated with lactation has, in previous case reports, been reported to be caused by a diet with low carbohydrate content [3, 4].

When the body’s glycogen storages are depleted, as under starvation, the brain’s demand for energy is met by gluconeogenesis and, later, due to low insulin levels, mobilization of free fatty acids into ketone bodies [5]; the little amount of glucose made through gluconeogenesis is used for galactopoiesis to the offspring.

Low carbohydrate diets became popular in the USA during the 1960s and peaked around year 2000, spurred by books written by Dr Stillman [6] and Dr Atkins [7]. In Sweden the low carbohydrate high fat (LCHF) diet was introduced in 2005 by a Swedish general practitioner and interest was generated when the creator started a blog, which criticized the traditional dietary advice for patients with diabetes [8]. Since then the diet has become increasingly popular.

## Case presentation

A 32-year-old white woman presented to our county hospital with a history of nausea, vomiting, heart palpitations, trembling and extremity spasms. She had started a strict LCHF diet, with an estimated carbohydrate intake of less than 20g per day, 10 days before admittance, lost 4 kilograms and had felt growing malaise. She was breastfeeding her son of 10 months of age. She continuously denied any alcohol or drug intake. She had a past medical history of hypothyreosis and had a family history of high blood pressure but not for diabetes. She took acetaminophen occasionally but no other medications.

The initial examination in the emergency department revealed an unaffected woman with respiratory rate of 12 breaths per minute, oxygen saturation 96% on room air, body temperature 36.3°C, pulse 102 beats per minute and blood pressure of 110/80mmHg. Nothing abnormal was revealed on examination of her heart, lungs, abdomen and thyroid gland.

An arterial blood gas was taken. It revealed pH 7.20, base excess (BE) −19, partial pressure of carbon dioxide (pCO_2_) 2.8 kPa, glucose 3.8mmol/l and lactate 1.0mmol/l. Her blood ketones were 7.1mmol/l (reference 0 to 0.5mmol/l). No genetic testing of any kind was performed.

The primary diagnosis was thought to be ketoacidosis due to starvation induced by the LCHF diet but blood samples for s-paracetamol, s-salicylate, s-ethanol, s-methanol, s-ethylene glycol, kidney function, diabetic autoantibodies, plasma cortisol (p-cortisol) and tests for thyroid function were added. She was admitted to our medical ward, given an intramuscular vitamin B injection and started on a 10% glucose infusion. In total 3L of glucose were infused, with an infusion rate of 125mL/hour, during 48 hours.

The following day, after glucose infusion and small doses of human insulin administered intravenously and insulin aspart subcutaneously, her acidosis was reversed. In total 4 units of insulin were administered during 24 hours. She was discharged, fully recovered, after 3 days and follow-up was performed after 1 month.

Diabetes mellitus was excluded by normal blood glucose and C-peptide, glycated hemoglobin (HbA1c) 37mmol/mol and negative autoantibodies: islet antigen number 2 (IA2) <15 kE/L, reference <15 and glutamic acid decarboxylase (GAD) <5 kE/L, reference <5. No drugs could be detected in her blood and her kidney function was normal (S-creatinine 67μmol/L, reference 45 to 90). The day after admittance her thyroid-stimulating hormone (TSH) was normal (2.5 mIE/L, reference 0.4 to 3.7) but her triiodothyronine (T_3_) and thyroxine (T_4_) were low: T_3_ 2.6pmol/L, reference 3.6 to 6.3 and T_4_ 11pmol/L, reference 12 to 22pmol/L. However, on the follow up 1 month later, her thyroid tests had normalized and thyroid peroxidase (TPO) antibodies were negative. Her p-cortisol was normal, 674nmol/L, in the morning (reference 200 to 800) as well as adrenocorticotropic hormone (ACTH). The lack of other possible explanations supported the primary diagnosis: ketoacidosis due to starvation.

## Discussion

In our case, a healthy lactating woman presented with ketoacidosis despite lack of a diabetes diagnosis. Ketoacidosis might on rare occasions be caused by a diet with low carbohydrate content. However, to the best of our knowledge this is the first reported case in the literature of ketoacidosis in a non-diabetic patient, associated with a combination of low carbohydrate, high fat diet and lactation. In previous cases of ketoacidosis, in lactating non-diabetic women as well as in other non-diabetic patients, physiological stressors such as fasting, infections, lactating twins and recently even bariatric surgery have been considered to be the push factor into ketoacidosis [9–14]. In our case the stressor seemed to be the LCHF diet in combination with lactation and its high demand of substrate to produce milk.

New diets are popular and with our modern communication, through Internet and blogs, the information spreads fast and is easily accessible. Authors on the Internet might be people supporting the diet or advertisers with commercial interests. It is a great challenge for medical services to be updated and to give proper information to our community.

It is important to make patients aware of the fact that active weight loss should not be undertaken during breast feeding, due to the high demand of substrate to produce milk and since the individual ability to utilize ketones usually is not known.

## Conclusions

A lactating woman has a high demand of substrate to produce milk. A LCHF diet limits the amount of substrate and results in a negative energy balance. This kind of diet should thus be avoided during lactation.

Our case shows that medical services should be aware of the fact that a strict LCHF diet often leads to ketosis and in rare cases even into ketoacidosis which is a dangerous condition that must be immediately diagnosed and treated in order to reduce morbidity.

## Consent

Written informed consent was obtained from the patient for publication of this case report. A copy of the written consent is available for review by the Editor-in-Chief of this journal.

## Abbreviations

LCHF: Low carbohydrate high fat
p-cortisol: Plasma cortisol
T_3_: Triiodothyronine
T_4_: Thyroxine
